# Mortality in the families of children during the COVID-19 pandemic: evidence from a repeated cross-sectional study of children in the United States

**DOI:** 10.1186/s12889-025-21528-7

**Published:** 2025-01-29

**Authors:** Kao-Ping Chua, Rena M. Conti, Nora V. Becker

**Affiliations:** 1 Susan B. Meister Child Health Evaluation and Research Center, University of Michigan Medical School, Ann Arbor, USA; 2 Department of Markets, Public Policy, Questrom School of Business, Boston University, And Law, Boston, USA; 3 Division of General Medicine, University of Michigan Medical School, Ann Arbor, USA; 4 2800 Plymouth Road, NCRC building 16, SPC 2800, Room G026W, Ann Arbor, Michigan, 48109-2800 USA

**Keywords:** COVID-19, Children, Mortality

## Abstract

**Background:**

Modeling studies suggest that hundreds of thousands of U.S. children have lost caregivers since the COVID-19 pandemic began. However, few studies have directly evaluated changes in mortality in the families of U.S. children using patient-level data, characterized adult decedents and their surviving children, or accounted for the loss of the adult siblings of children.

**Methods:**

We conducted a repeated cross-sectional analysis using Optum’s De-identified Clinformatics^®^ Data Mart, a national claims database that includes privately insured children and families across the U.S. We identified families of children aged 0–17 years enrolled in a family plan during each month in 2016–2021. Among these families, we calculated the proportion with ≥ 1 death of an adult family member aged ≥ 18 years enrolled in the plan, including young adult dependents aged 18–25 years. We used descriptive statistics to assess the age and sex of adult decedents and their surviving children. Moreover, we calculated the proportion of adult decedents who were the siblings of children.

**Results:**

From January 2016 through February 2020, the median monthly proportion of families with ≥ 1 adult death was 0.000076 (7.6 per 100,000 families). This rose to 12.1 per 100,000 families in December 2020, fell below the pre-pandemic median during March-July 2021, and exceeded this median for most months during August-December 2021. Among adult decedents, 63.8% were male and mean age was 44.8 (11.4) years. Among surviving children, 59.5% were adolescents aged 12–17 years. Among adult decedents, 4.8% were the siblings of children.

**Conclusions:**

During the pandemic, the proportion of privately insured children experiencing the loss of an adult family members varied over time, with the highest rates occurring at the end of 2020 and the second half of 2021. Some children lost adult siblings, suggesting that screening for child bereavement should include all family members, not just parents/guardians.

## Introduction

Modeling studies suggest that hundreds of thousands of U.S. children have lost caregivers since the COVID-19 pandemic began [1–4]. While important, these studies did not directly measure mortality using patient-level data but rather relied on projections. In addition, the studies did not characterize adult decedents and their surviving children or account for the loss of adult siblings.

In this analysis, we used 2016–2021 national commercial claims data to identify families of U.S. children enrolled in a private family insurance plan. We assessed trends in the monthly proportion of families experiencing the death of an adult family member enrolled in the plan, including young adult dependents. Moreover, we described the age and sex of adult decedents and their surviving children. Finally, we determined the proportion of adult decedents who were likely the parents/guardians versus older siblings of surviving children. Our findings could substantiate the results of modeling studies, generate new data on the demographic characteristics of adult decedents and their surviving children, and provide insights on whether bereavement of children during the pandemic may have been related to the deaths of adult siblings.

## Methods

### Data source

During spring-winter 2022, we conducted a repeated cross-sectional analysis of 2016–2021 data from Optum’s De-identified Clinformatics^®^ Data Mart, a national commercial claims database representing 12 million privately insured Americans annually. The database includes a family identifier that links patients in the same family plan. The database also includes information on month and year of death, which derives both from internal sources (e.g., plan disenrollment after death) and external sources (e.g., the Social Security Administration Death Master File [5]). Family relationships and cause of death are not reported. Although race/ethnicity data are reported, these data are frequently missing and are based on a proprietary algorithm that imputes race/ethnicity based on surname. Owing to these limitations, we do not report these data.

Because data are de-identified, the Institutional Review Board of the University of Michigan Medical School did not regulate this study as human subjects research; consequently, informed consent was not required. This manuscript follows the STROBE guidelines for observational studies.

### Study sample

We defined children as ages 0–17 years and adults as ages ≥ 18 years. We created 72 family-month cohorts, one for each month during 2016–2021. Families were included in a monthly cohort if ≥ 1 adult was enrolled at any point during the month and if ≥ 1 child was enrolled at any point and did not die during the month. We did not require continuous enrollment throughout the month for the child, as the death of an adult who was the primary plan holder could result in disenrollment of the entire family.

### Statistical analysis

For each cohort, we calculated the monthly proportion of families with ≥ 1 adult death, expressed as the number of families with ≥ 1 adult death per 100,000 families. We used quantile regression to compare the median of this proportion during the pre-pandemic period (January 2016-February 2020) and the pandemic period (March 2020-December 2021). We focused on medians rather than means owing to the wide variability in the monthly proportion of families with ≥ 1 adult death during the pandemic period.

Using descriptive statistics, we assessed the age and sex of adult decedents during the study period. We also compared the characteristics of adult decedents in the pre-pandemic and pandemic period using chi-squared tests (for categorical variables) and t-tests (for continuous variables).

Among adult decedents during the pandemic period, we used descriptive statistics to assess the age and sex of their surviving children. To infer the relationship between adult decedents and surviving children, we leveraged U.S. dependent eligibility rules in private family plans. We classified decedents aged ≥ 26 years as parents/guardians, as individuals cannot be dependents after age 25 [6]. We classified decedents aged 18–25 years as older siblings if the family plan also included ≥ 2 adults aged ≥ 40 years, a pattern consistent with the latter being the decedent’s parents/guardians. All other decedents aged 18–25 years were unclassified, including those in plans with only 1 adult aged ≥ 40 years, who could either be the decedent’s parent/guardian or spouse. Analyses used Stata 15.1/MP and two-sided hypothesis tests with α = 0.05.

### Sensitivity analysis

We repeated the comparison of the median monthly proportion of families with ≥ 1 adult death when defining the pandemic period as April 2020-December 2021, thus assigning March 2020 to the pre-pandemic period.

## Results

### Study sample

The sample included 80,089,783 family-months of data from 3,281,350 families, a mean (SD) of 24.4 (19.7) per family. Upon sample entry, 1,404,349 (42.9%) families resided in the South and mean family size was 3.6 (1.2) (Table 1).

**Table 1:** Characteristics of families in the sample overall and according to the occurrence and timing of mortality in adult family members

Characteristic^a^	All families in the sample	Families without any adult decedents^b^	Families with ≥ 1 adult decedent from January 2016-February 2020	Families with ≥ 1 adult decedent from March 2020-Dececember 2021
**Number of families**	3,281,350	3,275,174	4,381	1,795
**Family size**				
Mean (SD) no. family members	3.6 (1.2)	3.6 (1.2)	4.0 (1.3)	3.9 (1.3)
Mean (SD) no. adults	1.9 (0.8)	1.9 (0.8)	2.3 (1.0)^c^	2.2 (1.0)^c^
Mean (SD) no. children	1.7 (0.9)	1.7 (0.9)	1.7 (0.9)	1.7 (0.9)
**Age**				
Mean (SD) age of oldest adult	41.2 (8.3)	41.2 (8.3)	46.5 (8.7)	46.2 (8.4)
Mean (SD) age of oldest child	9.9 (5.3)	9.9 (5.3)	12.2 (4.4)	11.6 (4.5)
**Sex of oldest child**,** No. (%)**				
Female	1,604,918 (48.9%)	1,601,896 (48.9%)	2,139 (48.8%)	883 (49.2%)
Male	1,676,108 (51.1%)	1,672,954 (51.1%)	2,242 (51.2%)	912 (50.8%)
Unknown	324 (0.0%)	324 (0.0%)	0 (0.0%)	0 (0.0%)
**Region of residence**,** No. (%)**				
Northeast	294,230 (9.0%)	293,720 (9.0%)	378 (8.6%)	132 (7.4%)
Midwest	834,314 (25.4%)	832,534 (25.4%)	1,253 (28.6%)	527 (29.4%)
South	1,407,349 (42.9%)	1,404,601 (42.9%)	1,921 (43.8%)	827 (46.1%)
West	702,086 (21.4%)	700,965 (21.4%)	821 (18.7%)	300 (16.7%)
Unknown	43,371 (1.3%)	43,354 (1.3%)	8 (0.2%)	9 (0.5%)

^a^The table lists characteristics during the earliest month in which the family contributed data

^b^These were families for which there were no adult deaths during any month in which the family contributed data

^c^In most private insurance plans, patients aged 18–25 years can be covered as dependents on their parents’ insurance plan under the Affordable Care Act. Thus, covered adults can include more than just the plan holder and the plan holder’s spouse

### Trends in mortality among adult family members of children

**Fig. 1:**
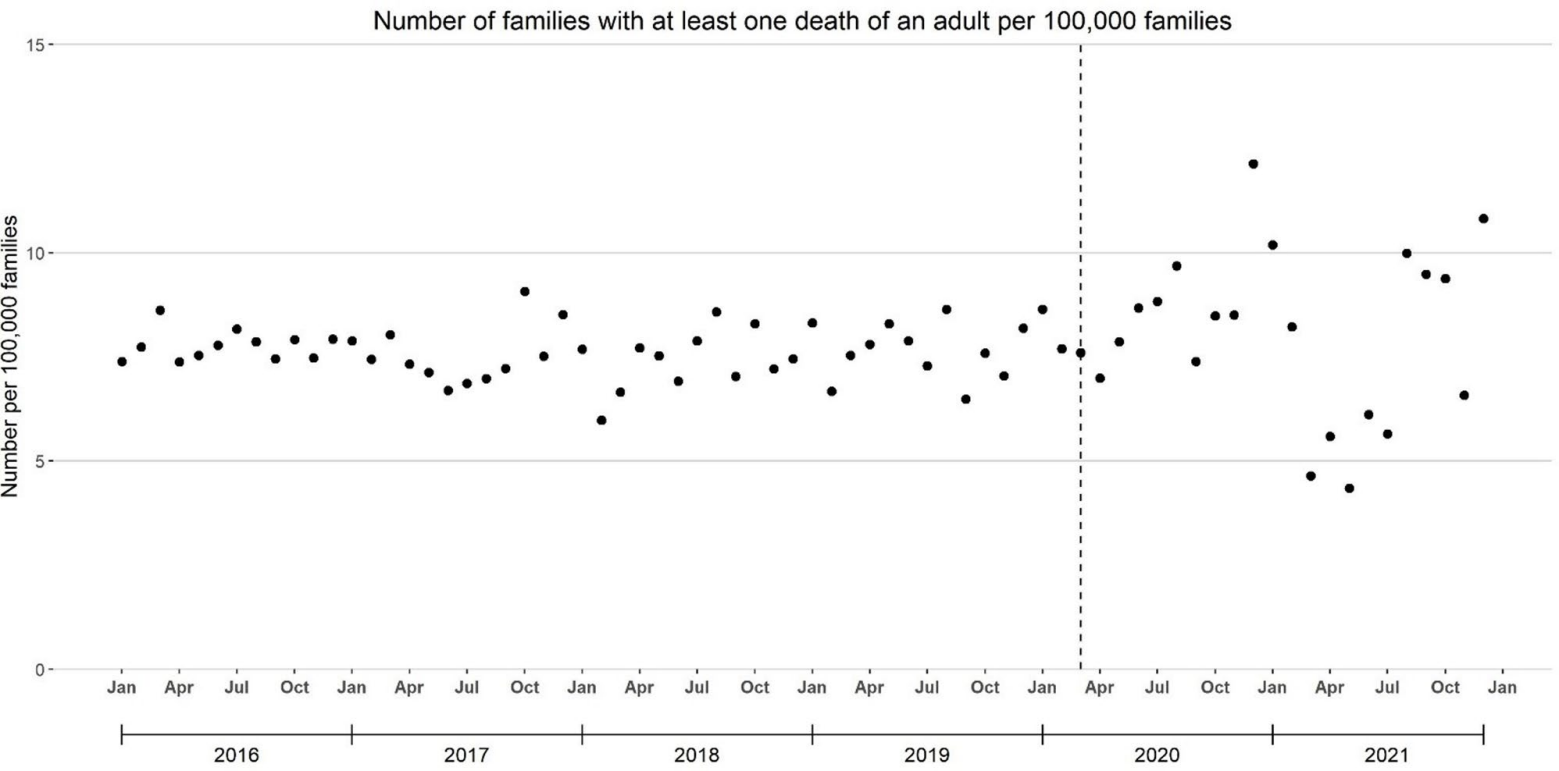
Monthly number of privately insured families of children with one or more death of an adult per 100,000 families, 2016–2021 Optum De-Identified Clinformatics^®^ Data Mart. The vertical line is March 2020, the beginning of the U.S. COVID-19 outbreak

During the pre-pandemic period, the median monthly proportion of families with ≥ 1 adult death was 0.000076 (7.6 per 100,000 families; 25th -75th percentile: 7.2–7.9 per 100,000 families). This rose to 12.1 per 100,000 families in December 2020 and decreased to 4.6–6.1 per 100,000 families from March through July 2021. Except for November 2021 (6.6 per 100,000 families), the median monthly proportion of families with ≥ 1 adult death exceeded the pre-pandemic median from August through December 2021 (range: 9.4–10.8 per 100,000 families) **(**Fig. 1**)**. Compared with the pre-pandemic period, the median monthly proportion of families with ≥ 1 adult death was higher (8.4 per 100,000 families, 25th -75th percentile: 6.6–9.5 per 100,000 families; IQR: 2.9), but this difference was not significant (*p* = 0.09).

### Characteristics of adult decedents and surviving children

During the study period, there were 6,216 adult decedents among 6,176 families. Among these decedents, 3,852 (62.0%) were male, 809 (13.0%) were aged 18–25 years, 489 (7.9%) were aged 26–34 years, 1,707 (27.5%) were aged 26–34 years, 2,264 (36.4%) were aged 45–54 years, 797 (12.8%) were aged 55–64 years, 97 (1.6%) were aged 65–69 years, and 53 (0.9%) were aged ≥ 70 years. Mean (SD) age was 43.2 (12.0) years.

As shown in Table 2, the sex distribution was similar among the 4,416 adult decedents during the pre-pandemic period and the 1,800 adult decedents during the pandemic period (*p* = 0.13). However, adult decedents during the pandemic periods were slightly older (mean age: 44.8 (11.4) versus 42.6 (12.2) years; *p* = 0.002). For example, 33 (0.8%) of adult decedents during the pre-pandemic period were aged ≥ 70 years, compared with 20 (1.1%) of adult decedents during the pandemic period.

**Table 2:** Characteristics of adult decedents overall and by period

Characteristic	All adult decedents (*n* = 6,216)	Adult decedents in the pre-pandemic period: Jan 2016-Feb 2020 (*n* = 4,416)	Adult decedents in the pandemic period: Mar 2020-Dec 2021 (*n* = 1,800)	*p* value^a^
Sex				
Female	2,363 (38.0)	1712 (38.8)	651 (36.2)	0.13
Male	3,852 (62.0)	2703 (61.2)	1149 (63.8)	
Unknown	1 (0.02)	1 (0.02)	0 (0.0)	
**Age**,** mean (SD)**	43.2 (12.0)	42.6 (12.2)	44.8 (11.4)	0.002
**Age group**				< 0.001
18–25 years	809 (13.0)	638 (14.5)	171 (9.5)	
26–34 years	489 (7.9)	365 (8.3)	124 (6.9)	
35–44 years	1707 (27.5)	1222 (27.8)	485 (26.9)	
45–54 years	2264 (36.4)	1560 (35.3)	704 (39.1)	
55–64 years	797 (12.8)	534 (12.1)	263 (14.6)	
65–69 years	97 (1.6)	64 (1.5)	23 (1.9)	
≥ 70 years	53 (0.9)	33 (0.8)	20 (1.1)	

^a^p value refers to the comparison between adult decedents in the pre-pandemic and pandemic periods. Chi-squared tests were used for categorical variables, and t-tests were used for continuous variables

Of the 1,800 adult decedents in the pandemic period, 1,629 (90.5%) were aged ≥ 26 years and classified as parents/guardians, 87 (4.8%) were classified as older siblings, and 84 (4.7%) were unclassified. The 1,800 adult decedents in the pandemic period were survived by 2,800 children. Of these children, 1,378 (49.2%) were male and 1,665 (59.5%) were aged 12–17 years.

### Sensitivity analysis

When assigning March 2020 to the pre-pandemic period, the median monthly proportion of families with ≥ 1 adult death during the pandemic period was significantly higher than during the pre-pandemic period (8.5 versus 7.6 per 100,000 families, *p* = 0.02), unlike in the main analysis.

## Discussion

In this national analysis of commercial claims data, the monthly proportion of privately insured U.S. families with ≥ 1 adult death rose sharply during winter 2020, fell below the pre-pandemic median during spring and early summer 2021, then rose above this median for most months during the second half of 2021. Overall, the median monthly proportion of families with ≥ 1 adult death was slightly higher during March 2020-December 2021 compared with January 2016-Feburary 2020. The difference was not significant in the main analysis, although the lack of significance was sensitive to the definition of the pandemic period.

The temporal pattern of deaths among the adult family members of children aligns with the surge in U.S. deaths from COVID-19 infection during the winter of 2020 and the second half of 2021 [7]. This finding indirectly suggests that many adult decedents may have died from COVID-19 infection. As further indirect evidence of this possibility, adult decedents in the pandemic period were slightly older than those in the pre-pandemic period, potentially owing to the increased lethality of COVID-19 infection in older populations [8].

The temporal pattern of deaths among the adult family members of deaths also aligns with modeling studies estimating the number of U.S. children experiencing the loss of one or both parents. For example, during the 3-month period between December 1, 2020, through February 28, 2021, one modeling study estimated that the number of such children increased from 54,675 to 102,468 (87.4%), compared with 36,808 through 54,175 (47.2%) in the prior 3-month period (September 1, 2020 through November 20, 2020) and 102,712 through 118,171 (15.1%) in the subsequent 3-month period (April 1, 2021 through June 30, 2021) [4]. In our study, we similarly found a sharp acceleration in the increase in the monthly proportion of children with ≥ 1 adult death during December 2020-February 2021, followed by a deceleration. The fact that our direct analyses of mortality patterns align with the projections from modeling studies increases confidence in the validity of the latter.

In our study, at least 90% of adult decedents during the pandemic period were likely the parents/guardians of surviving children, but at least 5% were likely older siblings aged 18–25 years. Moreover, among surviving children, approximately 6 in 10 were adolescents. These findings have at least two implications for clinicians. First, they suggest that child bereavement during the pandemic may sometimes have resulted from loss of adult siblings, not just parents/guardians. Screening for bereavement should therefore assess for loss of all family members. Second, findings suggest this screening may be particularly important for adolescents.

To our knowledge, this study represents the first national analysis to directly measure changes in mortality in the families of U.S. children during the COVID-19 pandemic, as well as the first to characterize adult decedents and their surviving children. Despite these contributions, our study had limitations. First, the database did not report cause of death. Consequently, we could not determine whether the observed increases in adult family member mortality during winter 2020 and late 2021 were driven by COVID-19 versus other causes, despite the fact that these increases coincided with overall rises in U.S. COVID-19 deaths, as noted above.

Second, our analysis may underestimate the proportion of privately insured families with ≥ 1 adult death, as adults outside of the family plan are not observed in the database and as the Social Security Administration Death Master File under-reports mortality [5]. However, any such underestimation would only bias our analysis of trends if it were differential between the pre-pandemic and pandemic periods.

Third, we lacked the statistical power to conduct subgroup analyses by geographic region or family characteristics. Finally, our database did not include publicly insured children, who may have been more likely than privately insured children to experience the loss of an adult family member owing to racial and ethnic disparities in mortality during the pandemic [9]. Consequently, our study likely underestimates increases in adult mortality in the families of all U.S. children after the COVID-19 outbreak. Unfortunately, linking children to parents in Medicaid claims databases is challenging. As such, future studies will likely need to address this limitation using other databases.

## Conclusion

Our findings suggest that screening for child bereavement should include all family members. Future research should continue to track changes in mortality in the families of U.S. children and assess the long-term health of child survivors.

## Data Availability

The data that support the findings of this study are available from Optum Labs but restrictions apply to the availability of these data, which were used under license for the current study, and so are not publicly available. Data are, however, available from the corresponding author (Dr. Chua) upon reasonable request and with permission of Optum Labs.
